# 3-(*p*-Tol­yl)-4-{3-[(phenyl­amino)­meth­yl]-7*H*-[1,2,4]triazolo[3,4-*b*][1,3,4]thia­diazin-6-yl}sydnone

**DOI:** 10.1107/S1600536811010786

**Published:** 2011-03-31

**Authors:** Hoong-Kun Fun, Ching Kheng Quah, Nithinchandra, Balakrishna Kalluraya

**Affiliations:** a X-ray Crystallography Unit, School of Physics, Universiti Sains Malaysia, 11800 USM, Penang, Malaysia; b Department of Studies in Chemistry, Mangalore University, Mangalagangotri, Mangalore 574 199, India

## Abstract

In the title compound, C_20_H_17_N_7_O_2_S (systematic name: 3-(4-methyl­phen­yl)-4-{3-[(phenyl­amino)­meth­yl]-7*H*-1,2,4-triazolo[3,4-*b*][1,3,4]thia­diazin-6-yl}-1,2,3-oxadiazol-3-ium-5-olate), the 3,6-dihydro-2*H*-1,3,4-thia­diazine ring adopts a half-boat conformation. The oxadiazol-3-ium ring makes dihedral angles of 57.99 (6) and 54.48 (6)° with the phenyl and benzene rings, respectively, while the 1,2,4-triazole ring forms corresponding angles of 37.35 (6) and 73.89 (6)°. The dihedral angle between the oxadiazol-3-ium and 1,2,4-triazole rings is 21.12 (6)°. In the crystal, the mol­ecules are linked *via* inter­molecular N—H⋯O and C—H⋯N hydrogen bonds into a layer parallel to the (100) plane. The crystal structure is further consolidated by C—H⋯π inter­actions. An intra­molecular C—H⋯O hydrogen bond is also observed, which generates an *S*(6) ring motif.

## Related literature

For general background to and the biological activity of sydnone derivatives, see: Rai *et al.* (2008 ▶); Kalluraya *et al.* (2002 ▶); Hedge *et al.* (2008 ▶). For general background to and the biological activity of triazolothia­diazine derivatives, see: Kalluraya & Rahiman (1997 ▶). For the synthesis of triazolothia­diazines, see: Kalluraya *et al.* (2003 ▶). For the stability of the temperature controller used in the data collection, see: Cosier & Glazer (1986 ▶). For bond-length data, see: Allen *et al.* (1987 ▶). For hydrogen-bond motifs, see: Bernstein *et al.* (1995 ▶). For ring conformations, see: Cremer & Pople (1975 ▶).
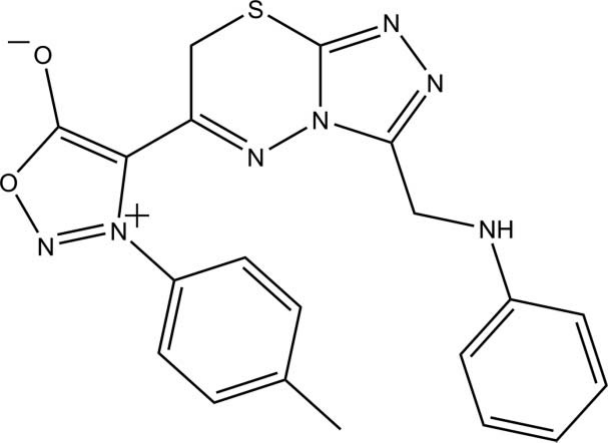


## Experimental

### 

#### Crystal data

C_20_H_17_N_7_O_2_S
*M* *_r_* = 419.47
Monoclinic, 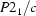

*a* = 10.1210 (4) Å
*b* = 10.5065 (4) Å
*c* = 19.6370 (6) Å
β = 114.550 (2)°
*V* = 1899.36 (12) Å^3^
*Z* = 4
Mo *K*α radiation
μ = 0.21 mm^−1^
*T* = 100 K
0.35 × 0.28 × 0.27 mm

#### Data collection

Bruker SMART APEXII DUO CCD area-detector diffractometer
Absorption correction: multi-scan (*SADABS*; Bruker, 2009 ▶) *T*_min_ = 0.932, *T*_max_ = 0.947
22363 measured reflections
6845 independent reflections
5754 reflections with *I* > 2σ(*I*)
*R*_int_ = 0.029

#### Refinement

*R*[*F* ^2^ > 2σ(*F* ^2^)] = 0.038
*wR*(*F* ^2^) = 0.109
*S* = 1.06
6845 reflections
272 parameters
H-atom parameters constrained
Δρ_max_ = 0.49 e Å^−3^
Δρ_min_ = −0.25 e Å^−3^

### 

Data collection: *APEX2* (Bruker, 2009 ▶); cell refinement: *SAINT* (Bruker, 2009 ▶); data reduction: *SAINT*; program(s) used to solve structure: *SHELXTL* (Sheldrick, 2008 ▶); program(s) used to refine structure: *SHELXTL*; molecular graphics: *SHELXTL*; software used to prepare material for publication: *SHELXTL* and *PLATON* (Spek, 2009 ▶).

## Supplementary Material

Crystal structure: contains datablocks global, I. DOI: 10.1107/S1600536811010786/is2692sup1.cif

Structure factors: contains datablocks I. DOI: 10.1107/S1600536811010786/is2692Isup2.hkl

Additional supplementary materials:  crystallographic information; 3D view; checkCIF report

## Figures and Tables

**Table 1:** Hydrogen-bond geometry (Å, °) *Cg*1 is the centroid of the C1–C6 phenyl ring.

*D*—H⋯*A*	*D*—H	H⋯*A*	*D*⋯*A*	*D*—H⋯*A*
C10—H10*A*⋯O2	0.97	2.40	3.1654 (14)	136
N1—H1⋯O2^i^	0.86	2.20	3.0218 (11)	160
C18—H18*A*⋯N2^ii^	0.93	2.62	3.4246 (14)	145
C15—H15*A*⋯*Cg*1^iii^	0.93	2.73	3.5537 (14)	148

Symmetry codes: (i) 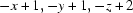; (ii) 
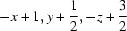
; (iii) 
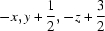
.

**Fig. 1.**
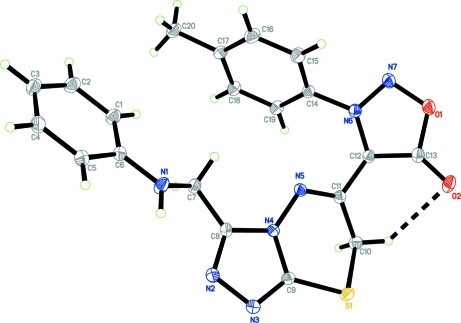
The molecular structure of the title compound showing 50% probability displacement ellipsoids for non-H atoms and the atom-numbering scheme. The intramoleculer hydrogen bond is shown as dashed line.

**Fig. 2.**
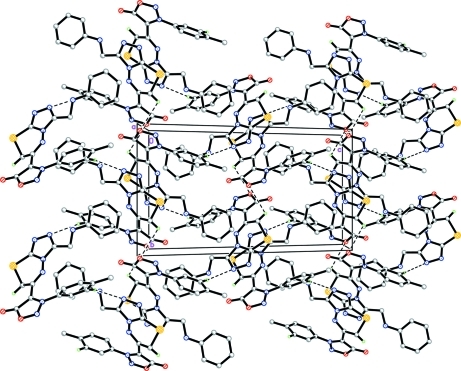
The crystal structure of the title compound, viewed along the a axis. H atoms not involved in hydrogen bonds (dashed lines) have been omitted for clarity.

## supplementary crystallographic information

### Comment

Sydnones are mesoionic heterocyclic aromatic compounds. The study of sydnones remains as a field of interest because of their electronic structures and varied types of biological activities displayed by some of them (Rai *et al.*, 2008). Recently, sydnone derivatives were found to exhibit promising antimicrobial properties (Kalluraya *et al.*, 2002). Triazolothiadiazines possess significant biological and pharmacological activities such as anthelmintic, analgesic and anti-inflammatory (Kalluraya & Rahiman, 1997) properties. Encouraged by these literatures, we have synthesized triazolothiadiazines containing the sydnone moiety. The introduction of sydnone moiety into an heterocyclic compound will increase the biological and pharmacological activities of heterocyclic system (Hedge *et al.*, 2008). Triazolothiadiazines were synthesized by the condensation of 4-bromoacetyl-3-arylsydnones with 3-aryloxymethyl- 4-amino-5-mercapto-1,2,4-triazoles. 4-Bromoacetyl-3-arylsydnones were in turn obtained by the photochemical bromination of 4-acetyl-3-arylsydnones (Kalluraya *et al.*, 2003).

The bond lengths (Allen *et al.*, 1987) and angles in the molecule (Fig. 1) are within normal ranges. The molecular structure is stabilized by an intramolecular C10–H10A···O2 hydrogen bond which generates an *S*(6) ring motif (Bernstein *et al.*, 1995). The 3,6-dihydro-2H-1,3,4-thiadiazine ring (S1/N5/N6/C9–C11) adopts a half-boat conformation with atom C10 deviating by 0.340 (1) Å from the mean plane through the remaining atoms, puckering parameters (Cremer & Pople, 1975) Q = 0.5312 (9) Å, Θ = 66.79 (11)° and φ = 325.26 (12)°. The dihedral angles between oxadiazol-3-ium ring (O1/N6/N7/C12/C13) and the two phenyl rings (C1–C6 and C14–C19) are 57.99 (6) and 54.48 (6)°, respectively. The correspondence angles for 1,2,4-triazole ring (N2–N4/C8/C9) are 37.35 (6) and 73.89 (6)°.

In the solid state (Fig. 2), the molecules are linked *via* intermolecular N1—H1···O2 and C18—H18A···N2 (Table 1) hydrogen bonds into infinite two-dimensional planes parallel to (100). The crystal structure is further consolidated by C15—H15A···*Cg*1 interactions (Table 1), where *Cg*1 is the centroid of C1–C6 phenyl ring.

### Experimental

To a solution of 4-bromoacetyl-3-(p-tolyl)sydnone (0.01 mol) and 4-amino-5-[(phenylamino)methyl]-4H-1,2,4-triazole-3-thiol (0.01 mol) in ethanol, catalytic amount of anhydrous sodium acetate was added. The solution was stirred at room temperature for 2 to 3 h. The solid product that separated out was filtered and dried. It was then recrystallized from ethanol. Crystals suitable for X-ray analysis were obtained from 1:2 mixtures of DMF and ethanol by slow evaporation.

### Refinement

H1 was located in a difference Fourier map and refined using a riding model with *U*_iso_(H) = 1.2 *U*_eq_(N). The remaining H atoms were positioned geometrically and refined using a riding model with C—H = 0.93–0.97 Å and *U*_iso_(H) = 1.2 or 1.5 *U*_eq_(C). A rotating-group model was applied for the methyl group. The highest residual electron density peak is located at 0.68 Å from C18 and the deepest hole is located at 1.01 Å from N4.

### Crystal data

**Table:**

C_20_H_17_N_7_O_2_S	*F*(000) = 872
*M**_r_* = 419.47	*D*_x_ = 1.467 Mg m^−^^3^
Monoclinic, *P*2_1_/*c*	Mo *K*α radiation, λ = 0.71073 Å
Hall symbol: -P 2ybc	Cell parameters from 8225 reflections
*a* = 10.1210 (4) Å	θ = 2.8–32.5°
*b* = 10.5065 (4) Å	µ = 0.21 mm^−^^1^
*c* = 19.6370 (6) Å	*T* = 100 K
β = 114.550 (2)°	Block, orange
*V* = 1899.36 (12) Å^3^	0.35 × 0.28 × 0.27 mm
*Z* = 4	

### Data collection

**Table:**

Bruker SMART APEXII DUO CCD area-detector diffractometer	6845 independent reflections
Radiation source: fine-focus sealed tube	5754 reflections with *I* > 2σ(*I*)
graphite	*R*_int_ = 0.029
φ and ω scans	θ_max_ = 32.6°, θ_min_ = 2.9°
Absorption correction: multi-scan (*SADABS*; Bruker, 2009)	*h* = −15→15
*T*_min_ = 0.932, *T*_max_ = 0.947	*k* = −15→15
22363 measured reflections	*l* = −29→29

### Refinement

**Table:**

Refinement on *F*^2^	Primary atom site location: structure-invariant direct methods
Least-squares matrix: full	Secondary atom site location: difference Fourier map
*R*[*F*^2^ > 2σ(*F*^2^)] = 0.038	Hydrogen site location: inferred from neighbouring sites
*wR*(*F*^2^) = 0.109	H-atom parameters constrained
*S* = 1.06	*w* = 1/[σ^2^(*F*_o_^2^) + (0.0589*P*)^2^ + 0.4436*P*] where *P* = (*F*_o_^2^ + 2*F*_c_^2^)/3
6845 reflections	(Δ/σ)_max_ = 0.002
272 parameters	Δρ_max_ = 0.49 e Å^−^^3^
0 restraints	Δρ_min_ = −0.25 e Å^−^^3^

### Special details

**Table:**

Experimental. The crystal was placed in the cold stream of an Oxford Cyrosystems Cobra open-flow nitrogen cryostat (Cosier & Glazer, 1986) operating at 100.0 (1) K.
Geometry. All esds (except the esd in the dihedral angle between two l.s. planes) are estimated using the full covariance matrix. The cell esds are taken into account individually in the estimation of esds in distances, angles and torsion angles; correlations between esds in cell parameters are only used when they are defined by crystal symmetry. An approximate (isotropic) treatment of cell esds is used for estimating esds involving l.s. planes.
Refinement. Refinement of F^2^ against ALL reflections. The weighted R-factor wR and goodness of fit S are based on F^2^, conventional R-factors R are based on F, with F set to zero for negative F^2^. The threshold expression of F^2^ > 2sigma(F^2^) is used only for calculating R-factors(gt) etc. and is not relevant to the choice of reflections for refinement. R-factors based on F^2^ are statistically about twice as large as those based on F, and R- factors based on ALL data will be even larger.

### Fractional atomic coordinates and isotropic or equivalent isotropic displacement parameters (Å^2^)

**Table:**

	*x*	*y*	*z*	*U*_iso_*/*U*_eq_	
S1	0.76994 (3)	0.59534 (2)	1.086823 (13)	0.01634 (7)	
O1	0.29144 (9)	0.96219 (7)	1.00427 (4)	0.01957 (15)	
O2	0.51181 (10)	0.92364 (7)	1.10036 (4)	0.02034 (16)	
N1	0.39442 (11)	0.30819 (8)	0.79980 (5)	0.01940 (18)	
H1	0.4424	0.2500	0.8313	0.023*	
N2	0.66867 (10)	0.35357 (8)	0.92354 (5)	0.01599 (16)	
N3	0.76192 (10)	0.39417 (8)	0.99600 (5)	0.01605 (16)	
N4	0.59144 (9)	0.53924 (8)	0.94311 (4)	0.01301 (15)	
N5	0.49010 (9)	0.63271 (8)	0.93523 (4)	0.01330 (15)	
N6	0.29928 (10)	0.84348 (8)	0.91836 (4)	0.01433 (15)	
N7	0.21520 (11)	0.92571 (9)	0.93095 (5)	0.01882 (17)	
C1	0.24653 (12)	0.35564 (10)	0.66818 (6)	0.01858 (19)	
H1A	0.2696	0.4417	0.6759	0.022*	
C2	0.15334 (12)	0.31226 (11)	0.59701 (6)	0.0203 (2)	
H2A	0.1146	0.3701	0.5578	0.024*	
C3	0.11761 (12)	0.18408 (11)	0.58390 (6)	0.0203 (2)	
H3A	0.0557	0.1559	0.5364	0.024*	
C4	0.17654 (12)	0.09824 (10)	0.64352 (6)	0.01943 (19)	
H4A	0.1535	0.0123	0.6355	0.023*	
C5	0.26881 (12)	0.13991 (10)	0.71441 (6)	0.01743 (18)	
H5A	0.3070	0.0817	0.7534	0.021*	
C6	0.30514 (11)	0.26966 (9)	0.72776 (5)	0.01523 (17)	
C7	0.44975 (12)	0.43689 (9)	0.81634 (5)	0.01550 (17)	
H7A	0.3723	0.4945	0.8128	0.019*	
H7B	0.4872	0.4638	0.7805	0.019*	
C8	0.56801 (11)	0.44026 (9)	0.89361 (5)	0.01385 (17)	
C9	0.71259 (11)	0.50470 (9)	1.00598 (5)	0.01399 (16)	
C10	0.68936 (11)	0.74328 (9)	1.03967 (6)	0.01621 (18)	
H10A	0.6906	0.8051	1.0766	0.019*	
H10B	0.7477	0.7769	1.0152	0.019*	
C11	0.53525 (11)	0.72606 (9)	0.98234 (5)	0.01326 (16)	
C12	0.42909 (11)	0.82264 (9)	0.97734 (5)	0.01385 (17)	
C13	0.42779 (12)	0.90140 (9)	1.03598 (5)	0.01612 (18)	
C14	0.24541 (11)	0.79366 (9)	0.84281 (5)	0.01405 (17)	
C15	0.10991 (12)	0.73640 (11)	0.81278 (6)	0.0201 (2)	
H15A	0.0557	0.7275	0.8409	0.024*	
C16	0.05717 (12)	0.69250 (12)	0.73922 (6)	0.0215 (2)	
H16A	−0.0334	0.6534	0.7180	0.026*	
C17	0.13799 (12)	0.70619 (10)	0.69677 (5)	0.01611 (18)	
C18	0.27547 (12)	0.76301 (10)	0.72997 (5)	0.01676 (18)	
H18A	0.3308	0.7710	0.7024	0.020*	
C19	0.33082 (12)	0.80769 (10)	0.80326 (5)	0.01638 (18)	
H19A	0.4219	0.8457	0.8251	0.020*	
C20	0.07796 (13)	0.66428 (11)	0.61610 (6)	0.0207 (2)	
H20A	0.0210	0.5886	0.6099	0.031*	
H20B	0.1567	0.6470	0.6022	0.031*	
H20C	0.0180	0.7305	0.5848	0.031*	

### Atomic displacement parameters (Å^2^)

**Table:**

	*U*^11^	*U*^22^	*U*^33^	*U*^12^	*U*^13^	*U*^23^
S1	0.01723 (12)	0.01541 (11)	0.01298 (10)	−0.00007 (8)	0.00287 (9)	−0.00047 (7)
O1	0.0246 (4)	0.0170 (3)	0.0176 (3)	0.0046 (3)	0.0092 (3)	−0.0020 (3)
O2	0.0298 (4)	0.0152 (3)	0.0137 (3)	0.0000 (3)	0.0068 (3)	−0.0018 (2)
N1	0.0258 (5)	0.0125 (3)	0.0133 (3)	−0.0004 (3)	0.0017 (3)	−0.0003 (3)
N2	0.0175 (4)	0.0158 (4)	0.0149 (3)	0.0018 (3)	0.0069 (3)	−0.0007 (3)
N3	0.0152 (4)	0.0168 (4)	0.0154 (3)	0.0015 (3)	0.0056 (3)	−0.0002 (3)
N4	0.0135 (4)	0.0120 (3)	0.0127 (3)	0.0014 (3)	0.0046 (3)	−0.0004 (3)
N5	0.0151 (4)	0.0114 (3)	0.0135 (3)	0.0021 (3)	0.0061 (3)	0.0004 (3)
N6	0.0169 (4)	0.0128 (3)	0.0136 (3)	0.0011 (3)	0.0066 (3)	0.0006 (3)
N7	0.0214 (4)	0.0178 (4)	0.0173 (4)	0.0049 (3)	0.0081 (3)	−0.0010 (3)
C1	0.0210 (5)	0.0182 (4)	0.0146 (4)	0.0002 (4)	0.0055 (4)	0.0007 (3)
C2	0.0192 (5)	0.0264 (5)	0.0143 (4)	0.0004 (4)	0.0058 (4)	0.0013 (4)
C3	0.0178 (5)	0.0278 (5)	0.0152 (4)	−0.0025 (4)	0.0067 (4)	−0.0050 (4)
C4	0.0186 (5)	0.0200 (4)	0.0203 (4)	−0.0017 (4)	0.0087 (4)	−0.0059 (3)
C5	0.0186 (5)	0.0156 (4)	0.0175 (4)	0.0009 (4)	0.0069 (4)	−0.0016 (3)
C6	0.0164 (4)	0.0157 (4)	0.0136 (4)	0.0008 (3)	0.0062 (3)	−0.0016 (3)
C7	0.0191 (5)	0.0137 (4)	0.0126 (4)	0.0004 (3)	0.0055 (3)	−0.0002 (3)
C8	0.0166 (4)	0.0128 (4)	0.0134 (4)	−0.0003 (3)	0.0074 (3)	−0.0010 (3)
C9	0.0123 (4)	0.0153 (4)	0.0136 (4)	0.0001 (3)	0.0046 (3)	0.0007 (3)
C10	0.0157 (4)	0.0137 (4)	0.0168 (4)	−0.0010 (3)	0.0043 (3)	−0.0010 (3)
C11	0.0157 (4)	0.0122 (4)	0.0124 (3)	0.0000 (3)	0.0064 (3)	0.0008 (3)
C12	0.0167 (4)	0.0118 (4)	0.0126 (3)	0.0008 (3)	0.0056 (3)	0.0001 (3)
C13	0.0225 (5)	0.0114 (4)	0.0153 (4)	0.0014 (3)	0.0087 (4)	0.0010 (3)
C14	0.0157 (4)	0.0140 (4)	0.0120 (3)	0.0011 (3)	0.0053 (3)	0.0008 (3)
C15	0.0167 (5)	0.0283 (5)	0.0175 (4)	−0.0025 (4)	0.0091 (4)	−0.0018 (4)
C16	0.0155 (5)	0.0306 (5)	0.0176 (4)	−0.0047 (4)	0.0061 (4)	−0.0026 (4)
C17	0.0161 (4)	0.0175 (4)	0.0132 (4)	0.0011 (3)	0.0046 (3)	0.0015 (3)
C18	0.0184 (5)	0.0190 (4)	0.0139 (4)	−0.0026 (4)	0.0078 (3)	0.0013 (3)
C19	0.0170 (4)	0.0177 (4)	0.0144 (4)	−0.0033 (3)	0.0066 (3)	0.0008 (3)
C20	0.0203 (5)	0.0254 (5)	0.0134 (4)	−0.0004 (4)	0.0040 (4)	−0.0004 (3)

### Geometric parameters (Å, °)

**Table:**

S1—C9	1.7315 (10)	C4—H4A	0.9300
S1—C10	1.8196 (10)	C5—C6	1.4079 (14)
O1—N7	1.3756 (12)	C5—H5A	0.9300
O1—C13	1.4093 (13)	C7—C8	1.4924 (14)
O2—C13	1.2177 (12)	C7—H7A	0.9700
N1—C6	1.3855 (12)	C7—H7B	0.9700
N1—C7	1.4479 (13)	C10—C11	1.5079 (14)
N1—H1	0.8621	C10—H10A	0.9700
N2—C8	1.3097 (13)	C10—H10B	0.9700
N2—N3	1.4075 (12)	C11—C12	1.4511 (14)
N3—C9	1.3106 (13)	C12—C13	1.4226 (13)
N4—C8	1.3751 (12)	C14—C15	1.3851 (15)
N4—C9	1.3790 (12)	C14—C19	1.3893 (14)
N4—N5	1.3814 (11)	C15—C16	1.3941 (15)
N5—C11	1.2943 (12)	C15—H15A	0.9300
N6—N7	1.3063 (12)	C16—C17	1.3968 (15)
N6—C12	1.3605 (13)	C16—H16A	0.9300
N6—C14	1.4491 (12)	C17—C18	1.4013 (15)
C1—C2	1.3983 (14)	C17—C20	1.5075 (14)
C1—C6	1.3999 (14)	C18—C19	1.3914 (13)
C1—H1A	0.9300	C18—H18A	0.9300
C2—C3	1.3904 (16)	C19—H19A	0.9300
C2—H2A	0.9300	C20—H20A	0.9600
C3—C4	1.3998 (16)	C20—H20B	0.9600
C3—H3A	0.9300	C20—H20C	0.9600
C4—C5	1.3861 (14)		
			
C9—S1—C10	95.43 (5)	N3—C9—N4	110.58 (8)
N7—O1—C13	110.89 (8)	N3—C9—S1	128.39 (8)
C6—N1—C7	121.96 (8)	N4—C9—S1	120.71 (7)
C6—N1—H1	117.3	C11—C10—S1	112.54 (7)
C7—N1—H1	116.9	C11—C10—H10A	109.1
C8—N2—N3	108.22 (8)	S1—C10—H10A	109.1
C9—N3—N2	106.42 (8)	C11—C10—H10B	109.1
C8—N4—C9	105.18 (8)	S1—C10—H10B	109.1
C8—N4—N5	123.82 (8)	H10A—C10—H10B	107.8
C9—N4—N5	129.05 (8)	N5—C11—C12	116.74 (9)
C11—N5—N4	115.69 (8)	N5—C11—C10	124.96 (9)
N7—N6—C12	114.83 (8)	C12—C11—C10	118.28 (8)
N7—N6—C14	115.79 (8)	N6—C12—C13	105.37 (9)
C12—N6—C14	129.26 (8)	N6—C12—C11	126.59 (8)
N6—N7—O1	104.84 (8)	C13—C12—C11	127.41 (9)
C2—C1—C6	120.04 (10)	O2—C13—O1	120.33 (9)
C2—C1—H1A	120.0	O2—C13—C12	135.62 (10)
C6—C1—H1A	120.0	O1—C13—C12	104.05 (8)
C3—C2—C1	121.08 (10)	C15—C14—C19	122.95 (9)
C3—C2—H2A	119.5	C15—C14—N6	118.54 (9)
C1—C2—H2A	119.5	C19—C14—N6	118.50 (9)
C2—C3—C4	118.79 (10)	C14—C15—C16	118.04 (10)
C2—C3—H3A	120.6	C14—C15—H15A	121.0
C4—C3—H3A	120.6	C16—C15—H15A	121.0
C5—C4—C3	120.77 (10)	C15—C16—C17	121.14 (10)
C5—C4—H4A	119.6	C15—C16—H16A	119.4
C3—C4—H4A	119.6	C17—C16—H16A	119.4
C4—C5—C6	120.51 (9)	C16—C17—C18	118.74 (9)
C4—C5—H5A	119.7	C16—C17—C20	121.11 (10)
C6—C5—H5A	119.7	C18—C17—C20	120.13 (9)
N1—C6—C1	122.34 (9)	C19—C18—C17	121.34 (9)
N1—C6—C5	118.82 (9)	C19—C18—H18A	119.3
C1—C6—C5	118.81 (9)	C17—C18—H18A	119.3
N1—C7—C8	108.80 (8)	C14—C19—C18	117.77 (10)
N1—C7—H7A	109.9	C14—C19—H19A	121.1
C8—C7—H7A	109.9	C18—C19—H19A	121.1
N1—C7—H7B	109.9	C17—C20—H20A	109.5
C8—C7—H7B	109.9	C17—C20—H20B	109.5
H7A—C7—H7B	108.3	H20A—C20—H20B	109.5
N2—C8—N4	109.59 (8)	C17—C20—H20C	109.5
N2—C8—C7	125.84 (9)	H20A—C20—H20C	109.5
N4—C8—C7	124.51 (9)	H20B—C20—H20C	109.5
			
C8—N2—N3—C9	−0.32 (11)	C9—S1—C10—C11	46.28 (8)
C8—N4—N5—C11	−172.93 (9)	N4—N5—C11—C12	−177.38 (8)
C9—N4—N5—C11	25.42 (14)	N4—N5—C11—C10	4.59 (14)
C12—N6—N7—O1	−1.21 (12)	S1—C10—C11—N5	−43.10 (12)
C14—N6—N7—O1	−177.58 (8)	S1—C10—C11—C12	138.89 (8)
C13—O1—N7—N6	1.44 (11)	N7—N6—C12—C13	0.52 (12)
C6—C1—C2—C3	−0.22 (17)	C14—N6—C12—C13	176.30 (9)
C1—C2—C3—C4	0.11 (17)	N7—N6—C12—C11	171.94 (9)
C2—C3—C4—C5	0.03 (17)	C14—N6—C12—C11	−12.28 (16)
C3—C4—C5—C6	−0.06 (17)	N5—C11—C12—N6	−17.48 (15)
C7—N1—C6—C1	−8.93 (16)	C10—C11—C12—N6	160.69 (9)
C7—N1—C6—C5	172.89 (10)	N5—C11—C12—C13	152.08 (10)
C2—C1—C6—N1	−177.99 (11)	C10—C11—C12—C13	−29.75 (15)
C2—C1—C6—C5	0.19 (16)	N7—O1—C13—O2	178.77 (9)
C4—C5—C6—N1	178.20 (10)	N7—O1—C13—C12	−1.14 (11)
C4—C5—C6—C1	−0.05 (16)	N6—C12—C13—O2	−179.49 (12)
C6—N1—C7—C8	−166.77 (10)	C11—C12—C13—O2	9.2 (2)
N3—N2—C8—N4	0.91 (11)	N6—C12—C13—O1	0.39 (10)
N3—N2—C8—C7	178.01 (9)	C11—C12—C13—O1	−170.93 (9)
C9—N4—C8—N2	−1.12 (11)	N7—N6—C14—C15	−55.54 (13)
N5—N4—C8—N2	−166.45 (9)	C12—N6—C14—C15	128.71 (11)
C9—N4—C8—C7	−178.27 (9)	N7—N6—C14—C19	123.23 (10)
N5—N4—C8—C7	16.41 (15)	C12—N6—C14—C19	−52.52 (14)
N1—C7—C8—N2	40.77 (14)	C19—C14—C15—C16	−0.75 (16)
N1—C7—C8—N4	−142.55 (10)	N6—C14—C15—C16	177.97 (10)
N2—N3—C9—N4	−0.40 (11)	C14—C15—C16—C17	−0.28 (17)
N2—N3—C9—S1	173.15 (8)	C15—C16—C17—C18	1.25 (17)
C8—N4—C9—N3	0.92 (11)	C15—C16—C17—C20	−177.10 (11)
N5—N4—C9—N3	165.20 (9)	C16—C17—C18—C19	−1.25 (16)
C8—N4—C9—S1	−173.20 (7)	C20—C17—C18—C19	177.12 (10)
N5—N4—C9—S1	−8.92 (14)	C15—C14—C19—C18	0.75 (15)
C10—S1—C9—N3	161.54 (10)	N6—C14—C19—C18	−177.96 (9)
C10—S1—C9—N4	−25.49 (9)	C17—C18—C19—C14	0.28 (15)

### Hydrogen-bond geometry (Å, °)

**Table:**

*Cg*1 is the centroid of the C1–C6 phenyl ring.

**Table:**

*D*—H···*A*	*D*—H	H···*A*	*D*···*A*	*D*—H···*A*
C10—H10A···O2	0.97	2.40	3.1654 (14)	136
N1—H1···O2^i^	0.86	2.20	3.0218 (11)	160
C18—H18A···N2^ii^	0.93	2.62	3.4246 (14)	145
C15—H15A···Cg1^iii^	0.93	2.73	3.5537 (14)	148

Symmetry codes: (i) −*x*+1, −*y*+1, −*z*+2; (ii) −*x*+1, *y*+1/2, −*z*+3/2; (iii) −*x*, *y*+1/2, −*z*+3/2.
